# 32-Phosphorus selectively delivered by listeria to pancreatic cancer demonstrates a strong therapeutic effect

**DOI:** 10.18632/oncotarget.15117

**Published:** 2017-02-06

**Authors:** Dinesh Chandra, Benson Chellakkan Selvanesan, Ziqiang Yuan, Steven K Libutti, Wade Koba, Amanda Beck, Kun Zhu, Arturo Casadevall, Ekaterina Dadachova, Claudia Gravekamp

**Affiliations:** ^1^ Albert Einstein College of Medicine, Department of Microbiology and Immunology, Bronx, NY 10461, USA; ^2^ Montefiore Medical Center, Medical Arts Pavilion, MMC-MAP, Bronx, NY 10467, USA; ^3^ Albert Einstein College of Medicine, Department of Radiology, MRRC, Bronx, NY 10461, USA; ^4^ Albert Einstein College of Medicine, Department of Pathology, Michael F. Price Center, Bronx, NY 10461, USA; ^5^ Johns Hopkins Bloomberg School of Public Health, Department of Molecular Microbiology and Immunology, Baltimore, MD 21205, USA; ^6^ Albert Einstein College of Medicine, Department of Radiology/Department of Microbiology and Immunology, Bronx, NY 10461, USA

**Keywords:** listeria monocytogenes, ^32^P, pancreatic cancer, KPC, Panc-02, delivery platform

## Abstract

Our laboratory has developed a novel delivery platform using an attenuated non-toxic and non-pathogenic bacterium *Listeria monocytogenes* that infects tumor cells and selectively survives and multiplies in metastases and primary tumors with help of myeloid-derived suppressor cells (MDSC) and immune suppression in the tumor microenvironment (TME). ^32^P was efficiently incorporated into the Listeria bacteria by starvation of the bacteria in saline, and then cultured in phosphorus-free medium complemented with ^32^P as a nutrient. Listeria-^32^P kills tumor cells through both ^32^P-induced ionizing radiation and Listeria-induced reactive oxygen species (ROS). The levels of ^32^P and Listeria were studied in various normal and tumor tissues, at sequential time points after injection of mice with pancreatic cancer (syngeneic model Panc-02). We found that ^32^P and Listeria predominantly accumulated in tumors and metastases, with their highest accumulation 4 hrs (^32^P) and 3 days (Listeria) after injection. Listeria also penetrated the transgenic KPC (conditionally express endogenous Kras-G12D and p53-R172H mutant alleles) pancreatic tumors and metastases. This is remarkable since KPC tumors, like human tumors, exhibit a stromal barrier, which prevents most drugs from penetrating the pancreatic tumors. Therapeutic treatment with Listeria -^32^P resulted in a strong reduction of the growth of pancreatic cancer at early and late stages in Panc-02 and KPC mice. These results highlight the power of Listeria as new delivery platform of anticancer agents to the TME. Not only were therapeutic levels of radioactive Listeria reached in tumors and metastases but the selective delivery also led to minimal side effects.

## INTRODUCTION

Poor delivery of anticancer agents to the tumor microenvironment (TME) is often one of the major struggles in cancer therapies. Pancreatic ductal adenocarcinoma (PDAC) is a good example because many therapies fail to penetrate the tumors due to a stromal barrier in the PDAC tumors [1, 2]. Moreover, metastases are particularly tough to treat by chemotherapy or small molecules because of inadequate target specificity and/or chemoresistance [3]. PDAC is characterized by its metastatic behavior before the primary tumor can be detected, resulting in a five-year survival rate of less than 5% [4, 5]. Gemcitabine and erlotinib, FDA-approved drugs for pancreatic cancer treatment, improve median survival by less than six months in advanced stage patients, underscoring the need for new alternative approaches [6]. One such approach could be anti-cancer therapy with attenuated *Listeria monocytogenes* (Listeria) bacteria. Our laboratory discovered that these bacteria selectively infect tumor cells and survive and multiply in tumors and metastases but not in healthy tissues [7, 8]. This is possible because Listeria infects myeloid-derived suppressor cells (MDSC) [7, 8], which are selectively attracted by the primary tumor through the production of attractants such as granulocyte macrophage-colony stimulating factor (GM-CSF), interleukin (IL)-6, or A100 [9, 10]. Once at the tumor site Listeria spreads from MDSC into tumor cells [7, 8] through a mechanism (polymerization of actin filaments and the production of Listeriolysin O) specific for Listeria [11], and kills the tumor cells through the generation of high levels of reactive oxygen species (ROS) [12]. Listeria also infects tumor cells directly [12]. They are protected from immune clearance in both the TME and MDSC because of their strong immune suppression, which is absent in healthy tissues [7, 8]. Based on these results we now use Listeria as a delivery platform for anticancer agents to the TME [7, 8, 13, 14]. In 2013, we demonstrated the power of the Listeria platform by accumulating high levels of 188Rhenium (^188^Re) through Listeria in the TME, resulting in a strong reduction of the pancreatic cancer [8]. This was the first time that a live attenuated bacterium was successfully used to deliver radioactivity selectively to metastases and tumors. In the current study, we developed a complete different and simplified method of generating radioactive Listeria (RL). Instead of coupling ^188^Re to Listeria with help of anti-Listeria antibodies we incorporated 32-Phosphorus (^32^P) directly into the Listeria by using 32P as a nutrient in the culture medium, without the need of antibodies. We found that Listeria and ^32^P accumulated in tumors and metastases but not healthy tissues. Listeria-^32^P appeared to be not only more effective than Listeria-^188^Re, but was also successful against late stage pancreatic cancer. Here, we provide data about a novel and simple method of Listeria-^32^P generation, its effect on early and advanced pancreatic cancer in Panc-02 and KPC mice, and its toxicity and potential for the treatment of patients with pancreatic and other cancers.

## RESULTS

### Generation and characterization of Listeria-^32^P

**Figure 1:**
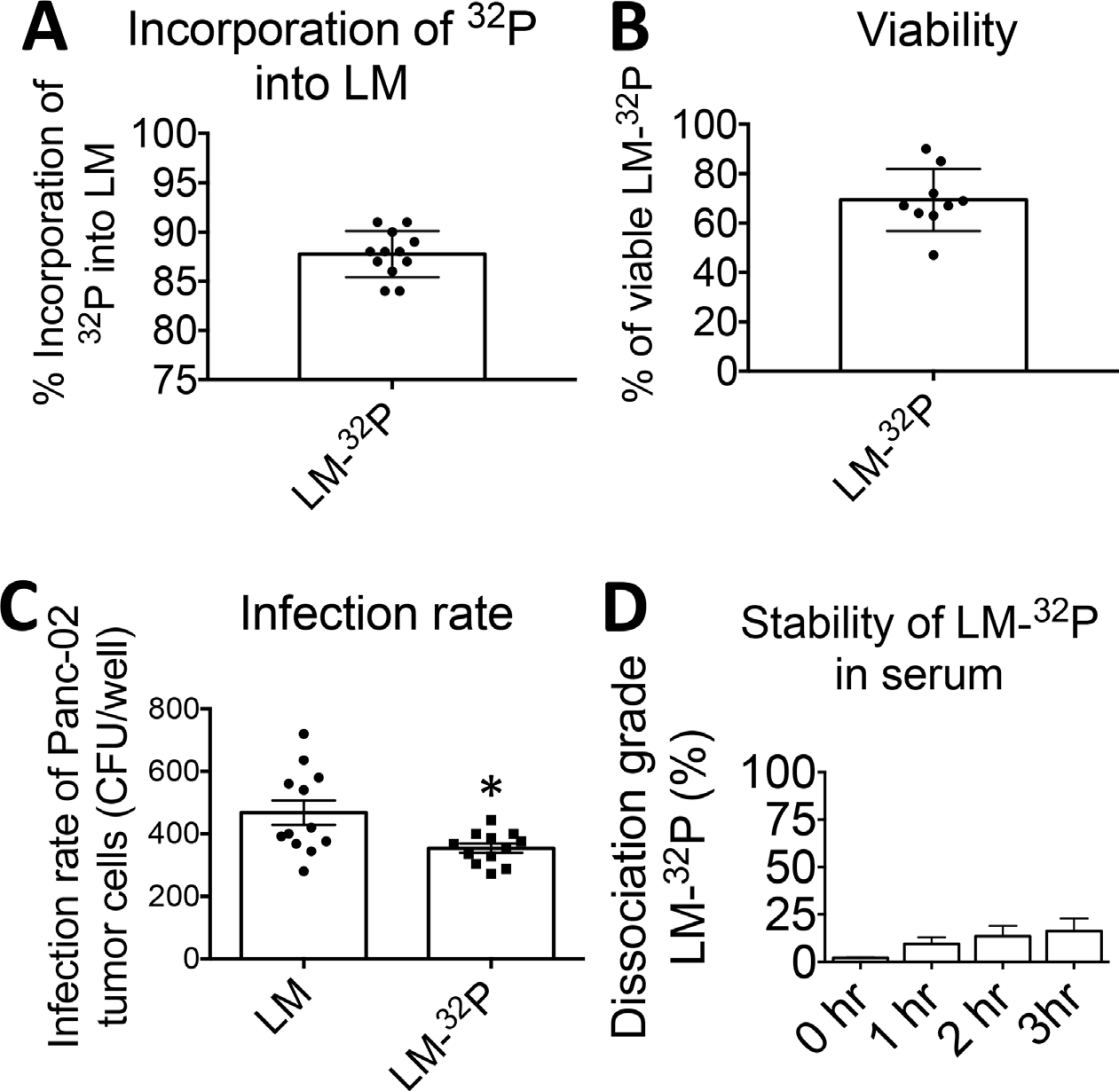
Generation and characterization of *Listeria monocytogenes* (Listeria)-^32^P (**A**) Incorporation of ^32^P into Listeria. Listeria was starved in saline and then cultured in phosphate free medium plus ^32^P. (Incorporation of ^32^P into Listeria = ^32^P in Listeria pellet/^32^P in Listeria pellet plus supernatant × 100%). (**B**) Viability of Listeria-^32^P. After incorporation of ^32^P into Listeria the viability of Listeria was compared to Listeria untreated (Viability of Listeria-^32^P = CFU of Listeria-^32^P/CFU of untreated × 100%). (**C**) Infection rate of tumor cells. Infection of Panc-02 tumor cells with Listeria-^32^P was compared to Listeria untreated. The number of CFU per well after 1 hr of infection was determined. The error bars represent SEM. Each dot represents one experiment. Unpaired *t* test *p = 0.0123 (**D**) Stability of Listeria-^32^P. The stability of Listeria-^32^P in serum was analyzed by incubating Listeria-^32^P with serum at 37°C. After 0, 1, and 3 h, the Listeria-^32^P bacteria were centrifuged, and the supernatant was analyzed for radioactive counts. The dissociation grade (percent) of the Listeria-^32^P complex was determined by dividing free radioactivity in the supernatant and baseline radioactivity (total radioactivity incorporated ^32^P into the Listeria plus free ^32^P in the supernatant). Data shown are the average of one experiment with *n* = 3 samples per time point. The error bars represent SEM.

First, the optimal conditions for ^32^P incorporation were determined, i.e. the highest incorporation of ^32^P and viability of the Listeria. For this purpose, Listeria bacteria (0.5 × 10^9^ CFU) were first starved in 1 ml of saline at 37°C, and then cultured in 1 ml of Edinburgh Minimal Media Phosphate Free (EMMP) medium complemented with ^32^P at 37°C. Various starvation times (30–120 min), incorporation times (30–120 min), and amounts of ^32^P (10–300 μCi) were tested. A reproducible and optimal incorporation protocol was developed consisting of 30 min of starvation of 0.5 × 10^9^ CFU of Listeria in 1 ml of saline, followed by 60 min culture in 1 ml of EMMP medium complemented with 50 μCi of ^32^P. We found that 93% of all the ^32^P was incorporated into the Listeria and that the results were highly reproducible (Figure 1A). We did not localize where the ^32^P was incorporated (cell wall, nucleus, cytoplasm, organelles), but it is expected that ^32^P incorporates at all place where phosphorus incorporates. Most importantly, we tested if ^32^P affected the viability of the Listeria.

To test the viability of Listeria, serial dilutions of untreated Listeria and Listeria-^32^P were made on agar plates and the number of CFU of Listeria was determined next day, as described previously [12]. The viability of the Listeria-^32^P was 69% (Figure 1B).

To test whether Listeria was still functional as a delivery platform for anticancer agents, we determined the infection rate of tumor cells with Listeria-^32^P. For this purpose, Panc-02 tumor cells were cultured with Listeria or Listeria-^32^P for 2 hrs, treated with Gentamicin to kill all extracellular bacteria, and then the Panc-02 tumor cells were lysed in water and plated on agar as described previously [8]. The number of Listeria CFUs was determined next day. A small but significant decrease in infection rate of Listeria-^32^P compared to Listeria was observed (Figure 1C).

Potentially, enzymes in the blood may dissociate ^32^P from Listeria. We confirmed that Listeria-^32^P reached the tumor and metastases before substantial dissociation of Listeria-^32^P could take place. Since it takes a few hours for ^32^P to accumulate in the TME (see below), we tested the dissociation grade of Listeria-^32^P in mouse serum after 0, 1, 2, and 3 hrs at 37°C. The dissociation grade after 3 hrs was 15% (Figure 1D).

In summary, a simple stable and reproducible method of generating Listeria-^32^P has been developed, without killing the Listeria itself (during one overnight), but delivering sufficient radioactivity to kill the tumor cells *in vivo* (see below).

### Accumulation of Listeria and 32P in tumors, metastases, and healthy tissues of Panc-02 model

**Figure 2:**
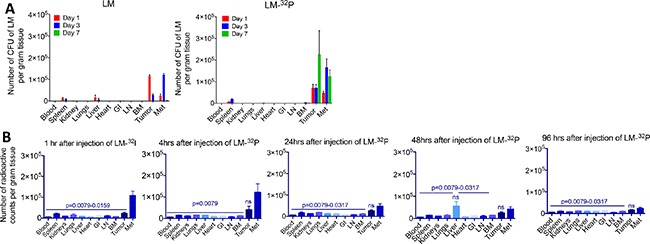
Effect of ^32^P on biodistribution of Listeria (**A**) Biodistribution of Listeria. Panc-02 mice were injected intraperitoneal (ip) with 0.5 × 10^7^ CFU of Listeria or Listeria-^32^P when tumors were 15 mm (day 14 after tumor cell injection), and 1, 3, and 7 days later the mice were euthanized and analyzed for the number of CFU of Listeria in tumors, metastases (predominantly in pancreas and portal liver, mesenteric lymph nodes along GI) and normal tissues as indicated on the x-axis. The error bars represent the SEM. Representative of 2 experiments. Data showing are the average of from *n* = 3 mice per group. (**B**) Biodistribution of ^32^P. Panc-02 mice were injected ip with 10^7^ CFU of Listeria-^32^P when tumors were 15 mm (day 10 after tumor cell injection), and 1, 4, 24, 48, and 96 hrs later the mice were euthanized and analyzed for radioactive counts (per gram tissue) in tumors, metastases and normal tissues as indicated on the x-axis. *n* = 5 mice per group. This is the average of one experiment. Mann-Whitney *p* < 0.05 is significant. All groups were compared to Listeria-^32^P. The error bars represent the SEM. ns = not significant. LN = lymph nodes, BM = bone marrow, Met = all metastases combined.

In a previous study we demonstrated that Listeria accumulated in tumors and metastases but not in normal tissues [8]. In the current study, we analyzed whether ^32^P affected the biodistribution of Listeria. For this purpose, we compared the number of CFU after injection of Listeria-^32^P with Listeria alone (Figure 2A). A similar distribution pattern was observed, i.e. in both cases Listeria accumulated in the tumors and metastases but not in normal tissues. However, one interesting difference was observed. The number of CFU of Listeria was higher after the injection of Listeria-^32^P than of Listeria in tumors and metastases, particular on day 7. On day 1 and 3 after injection of Listeria-^32^P or Listeria, the bacteria were found at higher numbers in the spleen and/or liver than in other normal tissues similar to the previous study [8] (but at much lower numbers than in tumors and metastases) (Figure 2A). This is partly because the spleen and/or liver are natural homing sites of Listeria. However, on days 14 (day 1 after injection of Listeria-^32^P) and 17 (day 3 after injection of Listeria-^32^P) after tumor cell injection, metastases already spread to the pancreas (in close proximity to the spleen) and liver. Therefore, in addition to the homing effect, the presence of metastases in the pancreas may have led to more Listeria bacteria in the spleen and liver than in other normal tissues.

To prove the selective delivery of ^32^P by Listeria to the tumors and metastases, we injected Panc-02 mice with Listeria-^32^P (10^7^ CFU delivering 1 μCi of radioactivity), and analyzed the radioactive counts at various time points after injection. The ^32^P uptake was the highest in metastases and tumors, particularly at 1 and 4 hrs after the injection (Figure 2B), while at 24, 48, and 96 hrs the ^32^P levels decreased but stayed significantly higher in tumors and metastases compared to the normal tissues, with an exception of the liver because metastases already spread to this organ. Also, liver is a natural homing site for Listeria and may have contributed to higher ^32^P levels compared to other normal tissues.

### Optimal dose of Listeria-^32^P and safety studies

**Figure 3:**
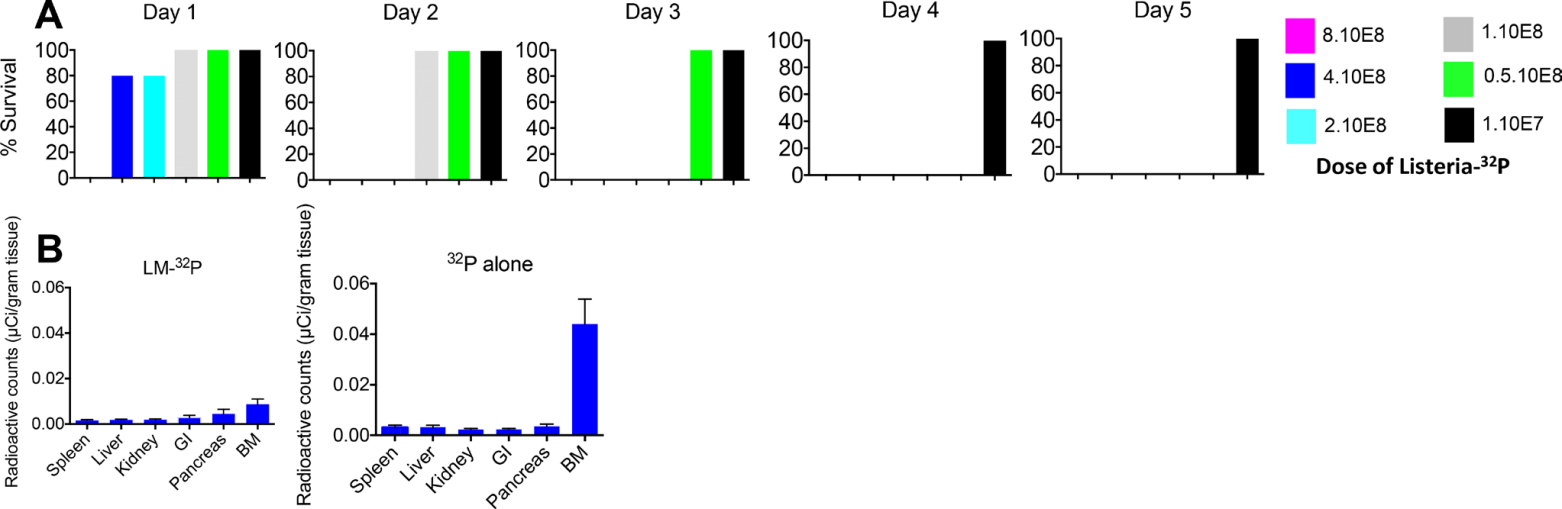
Safety of Listeria-^32^P (**A**) Dose limiting Toxicity (DLT) study. C57Bl6 mice (tumor-naïve) were injected ip with various doses of Listeria-^32^P, and analyzed for survival 0, 1, 2, 3, 4, and 5 days later. *n* = 5 mice per group. This is the average of one experiment. (**B**) Incorporation of ^32^P into bone marrow in C57Bl/6 mice without tumors. The mice received Listeria-^32^P or comparable amounts of ^32^P every day for 2 weeks. (10^7^ CFU of Listeria-^32^P represents 1 uCi of ^32^P). At the end of the 14 treatments all mice were euthanized and tissues were analyzed for radioactive counts using a scintillation counter, and normalized for μCi using a standard curve with known amounts (μCi) of ^32^P. ^32^P alone strongly incorporated into bone marrow (BM) cells, but not when delivered through Listeria. The error bars represent SEM. This experiment has been done once with *n* = 5 mice per group.

Increasing doses of Listeria-^32^P were injected in C57Bl/6 mice without cancer and the survival time was determined over the course of the next 5 days. In a previous study we found that the LD_50_ of Listeria alone is 10^8^ CFU [15). However, since Listeria is incorporated with ^32^P we tested the LD_50_ more narrowly. The following doses were tested: 1 × 10^7^, 0.5 × 10^8^, 1 × 10^8^, 2 × 10^8^, 4 × 10^8^, and 8 × 10^8^ CFU of Listeria-^32^P. As shown in Figure 3A, 107 CFU was the optimal dose because none of the mice died, similar as we found with the Listeria alone [15]. However, all mice died on day 4 when injected with 5 × 10^7^ CFU, and 10^8^ CFU and higher resulted in mice dying on day 3 and thereafter. The LD_50_ of Listeria-^32^P is most likely around 2.5 × 10^7^, but this needs to be confirmed. In addition, we analyzed tumor-naïve mice injected with Listeria-^32^P by pathological examination one month after treatment with 12 doses of 10^7^ CFU of Listeria-^32^P, and no serious pathological damage was observed (Supplementary Table 1). We also analyzed potential toxicity by pathological examination, 3 months after the last treatment with 12 doses of 10^7^ CFU of Listeria-^32^P in the Panc-02 mice. Again, no pathological damage could be observed (Supplementary Table 2). In summary, these results indicate that the dose of 10^7^ CFU of Listeria-^32^P appeared to be the optimal dose for the treatment of pancreatic cancer in Panc-02 mice.

Finally, we analyzed whether ^32^P incorporated into bone marrow cells, because this is often reported for ^32^P. After 12 doses with 10^7^ CFU of Listeria-^32^P, ^32^P was barely incorporated into bone marrow (BM), but when ^32^P was administered as a single agent its incorporation level was much and significantly higher compared to Listeria-^32^P (Figure 3B).

### Efficacy and survival studies in Panc-02 mice

**Figure 4:**
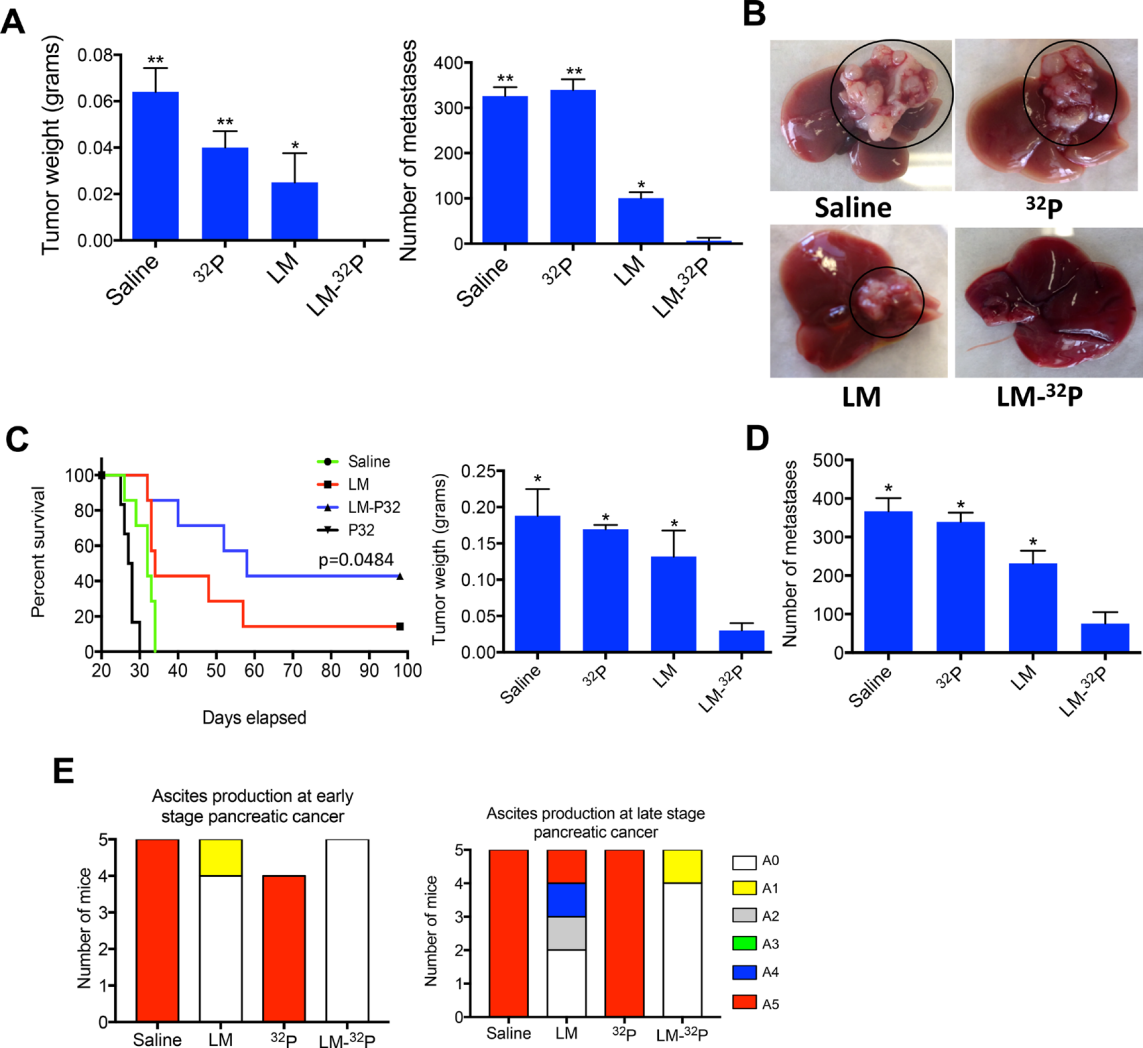
Testing Listeria-^32^P in pancreatic cancer model Panc-02 (**A**) Listeria-^32^P is highly effective against early stage pancreatic cancer. C57Bl/6 mice were injected with 2 × 10^6^ Panc-02 cells in the mammary fat pad, and subsequently injected ip with 10^7^ CFU of Listeria-^32^P (delivering 1 μCi ^32^P), 10^7^ CFU of Listeria, 1 μCi ^32^P, or saline, every 3 days for 2 weeks (starting at day 3 after tumor cell injection). A total of 6 uCi was delivered by Listeria in this experiment. One week after last treatment (day 28) mice were analyzed for tumor weight and frequency of metastases (liver, pancreas, mesenteric lymph nodes, spleen, kidneys). Untreated mice will die around day 30. Mann-Whitney *p* < 0.05 is significant. All groups were compared to Listeria-^32^P. The error bars represent the SEM. Representative of two experiments. (**B**) Example of metastases in liver of Panc-02 mice treated with Listeria-^32^P, Listeria, ^32^P or Saline. Representative of two experiments with *n* = 5 mice per group. An example of 5 livers in each group of one experiment is displayed in the Supplementary Figure 1. (**C**) Listeria-^32^P strongly improves survival rate of panc-02 mice. Mice were injected with 2 × 10^6^ Panc-02 tumor cells into the mammary fat pad, and 12 doses of 10^7^ CFU of Listeria-^32^P (delivering 1 μCi of ^32^P), were started 3 days after tumor cell injection. The mice were monitored up to day 100 after tumor cell injection. All groups were compared to the saline group. The survival curves were analyzed by Log-rank (Mantel-Cox) test. *p* > 0.05 is statistically significant. This experiment has been done once with *n* = 6–7 mice per group. (**D**) The effect of Listeria-^32^P on pancreatic cancer in advanced stage (Panc-02 model). Mice were injected with 10^5^ Panc-02 tumor cells in the mammary fat pad. When tumors reached 8–10 mm (10 days after tumor cell injection), and metastases had spread to all organs, the Panc-02 mice received three cycles of 10^7^ CFU of LM-^32^P or saline on four consecutive days, followed by a rest period of three days after each cycle (12 doses total). The mice were euthanized 6 weeks after tumor cell injection. *n* = 5 mice per group. The results were averaged. This experiment was performed once. Mann-Whitney *p* < 0.05 is significant. The error bars represent the SEM. (**E**) Ascites production in treated and untreated Panc-02 mice with early and advanced pancreatic cancer. At the end of treatments, the production of ascites was graded in the peritoneal cavity in each mouse by visual inspection using a scale from A0 to A5 (A0 = no production, A5 = highest production of ascites), as described in Materials and Methods.

We then performed an efficacy study with Listeria-^32^P in Pan-02 mice aiming to kill the tumor cells through Listeria-induced ROS and ^32^P. C57Bl/6 mice were injected with 2 × 10^6^ tumor cells in the mammary fat pad and three days later, the mice were treated with 10^7^ CFU of Listeria, every 3 days for 2 weeks (6 doses totally). At the end of the treatments (day 28), primary tumors were undetectable by eye in 100% of the mice and just a few metastases were detected in 20% of the mice that received Listeria-^32^P (Figure 4A, 4B and Supplementary Figure 1), while in the saline and ^32^P groups, tumors and metastases as well as ascites were detected in 100% of the mice (Figure 4A, 4B). Also in the group treated with Listeria alone, 100% of the mice developed tumors and metastases, but significantly less than in the saline and ^32^P groups. To determine whether the mice that received Listeria-^32^P were really cancer free, a survival study was performed (and further improved the treatment protocol). Tumor-bearing mice received twelve doses 10^7^ CFU of Listeria-^32^P or Listeria alone, or twelve doses of ^32^P alone. We found that 43% of the mice that received Listeria-^32^P was free of cancer (confirmed by pathological examination; Supplementary Table 2), and that the other 57% of the mice treated with Listeria-^32^P lived twice as long as mice treated with Saline or ^32^P alone (Figure 4C). The Listeria alone was also effective but significantly less than the Listeria-^32^P. Early treatment with ^32^P alone had also some effect on tumors, because free ^32^P evenly spreads over normal and tumor tissues. However, free ^32^P is toxic and therefore resulted in early deaths, even before mice in the saline group died.

Most patients are diagnosed when the pancreatic cancer has advanced with metastases already spread to the liver and other organs. Therefore, we performed a study to analyze the effect of Listeria-^32^P on advanced pancreatic cancer, i.e. we now injected 10^5^ tumor cells (because untreated mice with 2 × 10^6^ tumor cells die within 28 days) and treatments (12 doses of 10^7^ CFU of Listeria-^32^P) were started on day 10 after tumor cell injection (when tumors were 8–10 mm), and mice were euthanized 6 weeks later. As shown in the Figure 4D, Listeria-^32^P was strongly effective against tumors and metastases when started the treatment at advanced stage. Tumor weight and number of metastases were significantly reduced by Listeria-^32^P compared to all control groups. However, at this stage of the pancreatic cancer, Listeria alone had no significant effect on tumors or metastases (Supplementary Figure 2).

All untreated Panc-02 mice developed ascites in the peritoneal cavity. Listeria-^32^P strongly reduced the production of ascites in Panc-02 mice with early and advanced pancreatic cancer (Figure 4E). LM-^32^P completely prevented the production of ascites in Panc-02 mice with early pancreatic cancer and almost completely in Panc-02 mice with advanced pancreatic cancer (20% of the mice produced some ascites graded as A1).

### Penetration of Listeria into KPC tumors and selective accumulation of Listeria in the KPC tumors

**Figure 5:**
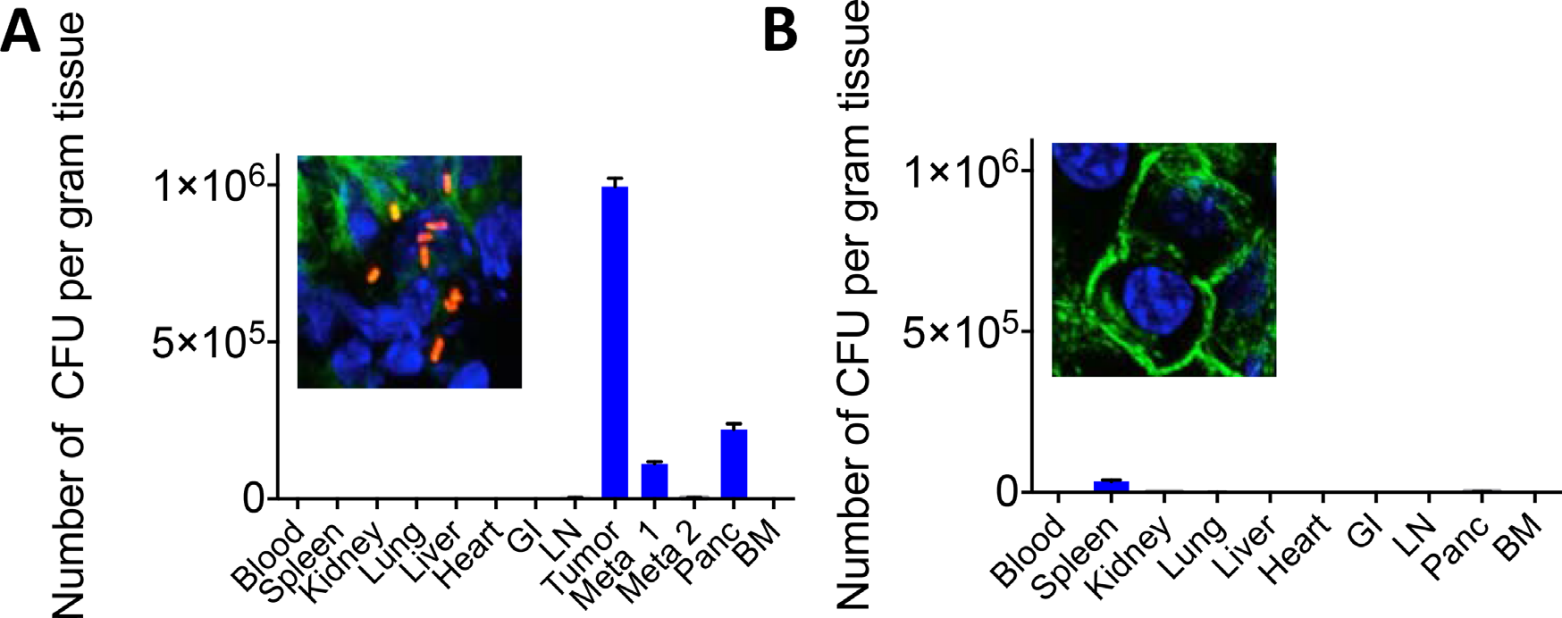
Penetration of Listeria into pancreatic tumor, metastases and healthy tissues of KPC model KPC mice received one dose of 10^7^ CFU of Listeria ip. Two days later, the number of CFU of Listeria was measured in all tissues, including the pancreatic tumor and metastases. (**A**) KPC mouse with late stage pancreatic cancer. (**B**) KPC mouse with early stage pancreatic cancer (tumor and metastases not detectable yet). Confocal microscopy pictures show the pancreas with or without Listeria of KPC mice with or without pancreatic cancer, respectively.

One of the main reasons for the failure of gemcitabine and other drugs in patients with PDAC is a barrier of stromal cells that prevents the efficient penetration of the chemotherapeutic drugs into the pancreatic tumors [1, 16]. KPC mice, a transgenic mouse tumor model with PDAC (conditionally express endogenous Kras-G12D and p53-R172H mutant alleles) is known for its stromal barrier in the pancreatic tumors, and develops multiple metastases in the liver and lung (1), similar to pancreatic cancer patients. We injected the KPC mice of 6 months (tumors and metastases present) or KPC mice of 6 weeks (no visible malignancies) with 10^7^ CFU of Listeria, and two days later the Listeria bacteria were isolated from healthy tissues, tumors and metastases. Listeria bacteria were abundantly found in the tumors and metastases and much less or not at all in healthy tissues of the 6-month old KPC mice, and also not or much less in all tissues of the 6-weeks old KPC mice without visible malignancies (Figure 5A, 5B). This was confirmed by confocal microscopy. In summary, Listeria bacteria were able to penetrate the KPC tumors and metastases abundantly, while no or much less Listeria bacteria were found in healthy tissues or in potential preneoplastic tissues in the pancreas of the young KPC mice.

### Efficacy study of Listeria-^32^P in KPC mice

**Figure 6:**
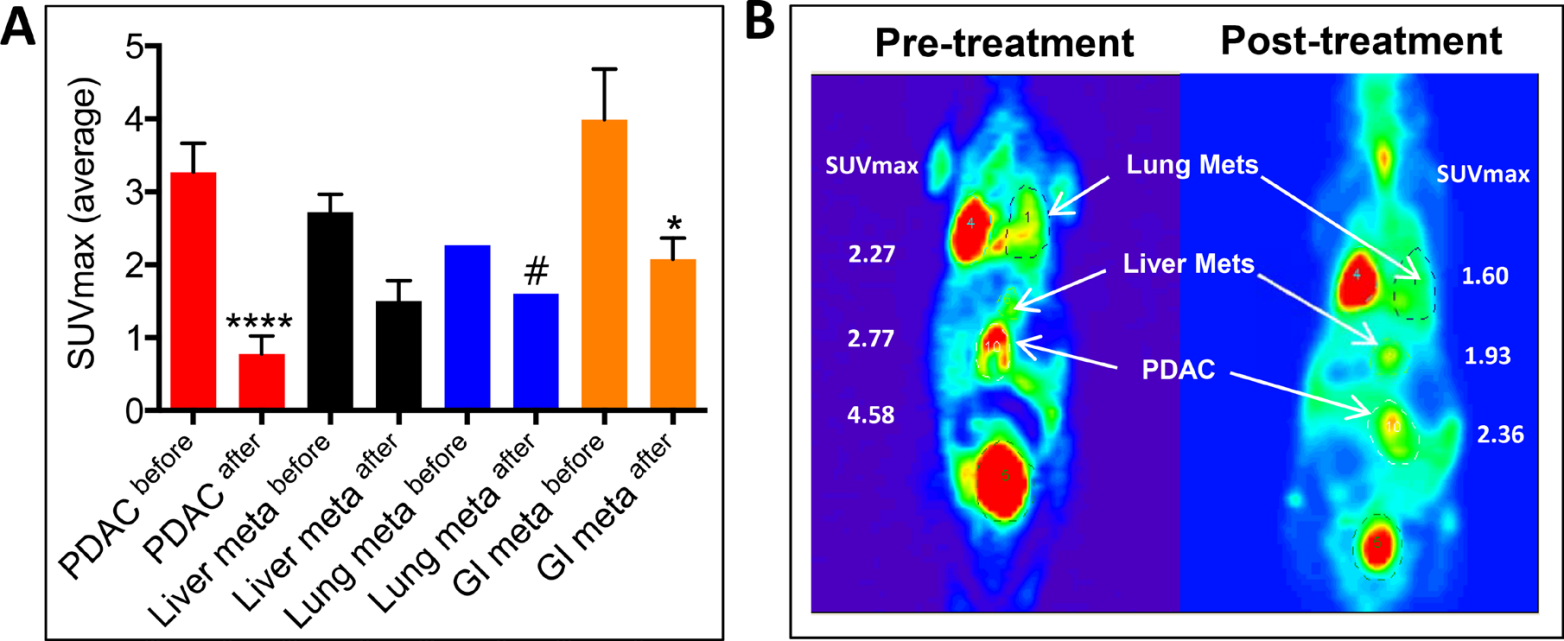
Listeria-^32^P reduces grow of tumor and metastases (liver and lungs) in KPC mice with advanced pancreatic cancer KPC mice received three cycles of Listeria-^32^P ip for four consecutive days with 3 days rest between each cycle (12 doses in total). (**A**) The effect of Listeria-^32^P treatment (SUVmax) was monitored before and after treatment by PET or PET/CT. The results were averaged and subjected to statistical analysis Mann-Whitney *p* < 0.05 is significant. The error bars represent SEM. *n* = 9 KPC mice. (**B**) Example of Listeria-^32^P treatment monitored before and after Listeria-^32^P by PET/CT in KPC mouse.

Finally, we tested the effect of Listeria-^32^P on tumors and metastases in the KPC mice with advanced stages of pancreatic cancer. Three cycles of 10^7^ CFU of Listeria-^32^P were injected for four consecutive days (12 doses in total) with three days rest at the end of each cycle. Effect of treatments was monitored before and after treatment by positron emission tomography/computed tomography (PET/CT) or PET alone. This analysis was used because it mimics the analysis of treatment in cancer patients most closely. Effects of ^32^P and Listeria alone already have been analyzed in the Panc-02 model. As shown in Figure 6A, before treatment the KPC mouse exhibits a large tumor in the pancreas (PDAC) and metastases in the lungs and liver. Listeria-^32^P treatment reduced the growth of the PDAC tumors and metastases along the gastro intestines (GI) in mesenteric lymph node areas (SUVmax) significantly (85% and 67%, respectively), while the number of KPC mice with metastases in lungs (1/9) and liver (3/9) were insufficient to demonstrate significance. Even with an extremely large PDAC tumor (largest diameter 2–3 cm; SUVmax before treatment: 6.75 and SUVmax after treatment: 6.35), Listeria-^32^P was still able to prevent its further progression. An example of PET/CT is shown in Figure 6B. All tumors and metastases were confirmed by pathological examination.

We also analyzed the SUVmax in KPC mice with advanced pancreatic cancer (8 months of age) before and after Listeria treatment (control group), and compared to non-treated and Listeria-treated Panc-02 mice with advanced pancreatic cancer. Similar to Panc-02 mice with advanced pancreatic cancer, no significant effect of Listeria alone was observed in the KPC mice (Supplementary Figure 2).

## DISCUSSION

In the 1890s William Coley injected live bacteria into tumors aiming to boost the immune system against host's own tumors. However, these bacteria were not attenuated and some patients died because of the treatment while others did better than the non-treated patients and their tumors disappeared. We used an attenuated non-toxic and non-virulent *Listeria monocytogenes* to deliver anticancer agents to the TME. After discovering that Listeria could selectively infect tumor cells with help of MDSC, and survive and multiply in the TME we started using Listeria as a selective delivery platform for anticancer agents [8, 14]. In the study presented here, we developed a new method of generating radioactive Listeria by simply incorporating the ^32^P directly into the Listeria bacteria during culture. After injection of Listeria-^32^P into mice with pancreatic cancer, we showed that Listeria and ^32^P selectively accumulated in tumors and metastases but not in healthy tissues. Listeria-^32^P showed a great effect on metastases and tumors in both pancreatic cancer models Panc-02 and KPC at early and advanced stages of pancreatic cancer, while Listeria alone showed a significant effect on early pancreatic cancer (Panc-02 mice) but not on advanced pancreatic cancer.

In 2009, we reported that LLO induced high levels of ROS through the activation of the NADPH-oxidase pathway, resulting in tumor cell kill through DNA damage [12]. Listeria infection was reported to induce DNA damage in HELA cells, with the mechanism involving LLO blocking of the signaling response to DNA breaks through degradation of Mre11 (sensor of DNA damage), which improved replication of the Listeria bacteria [17]. Since ^32^P-induced ROS also induces DNA breaks, the LLO-induced decrease in DNA damage responses (DDR), may promote replication of Listeria-^32^P more than Listeria alone in the TME. If so, this could also happen in normal tissues. We found that the number of CFU of Listeria-^32^P (Figure 2A) was higher than the CFU of Listeria alone [8] in tumors and metastases, particularly on day 7, supporting the results of Cossart's group. However, this was not observed in normal tissues, i.e. Listeria-^32^P was present at very low numbers or undetectable on day 7 after injection of Listeria-^32^P. Most likely, immune responses have eliminated the Listeria-^32^P in normal tissues efficiently, and consequently eliminated the possibility to promote replication of the Listeria in normal tissues induced by LLO-decreased DDR responses. Also, the ^32^P levels in normal tissues may have been too low (Figure 2B) to promote replication of Listeria. Of note is that the Cossart group used wild type Listeria that is known to replicate in the liver and in epithelial cells of the GI [18] and that most of their experiments were performed *in* *vitro* where immune responses are absent. In summary, ^32^P and Listeria may have a synergistic effect on tumor cell death but immune responses may have prevented to have this effect on normal cells. Of note is that 1 and 3 months after 12 Listeria-^32^P treatments no toxic effects were observed in normal tissues. It would be interesting to study mechanism(s) of potential synergistic effect(s) of Listeria and ^32^P in the TME and normal tissues in more detail.

Most therapies against pancreatic cancer fail because primary tumors are surrounded by a stromal barrier of fibrotic cells that prevent penetration of the drug into the primary tumors [1, 2]. We have shown that Listeria easily penetrates the primary tumor, which is most likely because Listeria uses MDSC as a delivery vehicle [7, 8]. In this regard, both human and mouse PDAC are heavily infiltrated with MDSC [1, 19].

Incorporation of ^32^P into bone marrow cells is one of the main concerns for clinicians. Indeed, we found that free ^32^P did incorporate into the bone marrow cells, but interestingly when delivered through Listeria the incorporation into bone marrow cells was minimal. It is possible that ^32^P is not available for incorporation into bone marrows cells when already incorporated into the Listeria. Alternatively, the Listeria-^32^P is eliminated by the immune system before it reaches the bone marrow. To further analyze potential toxicity of the Listeria-^32^P, extensive pathological examinations and toxicity studies were performed (Supplementary Tables 1 and 2). No side effects of the Listeria-^32^P were observed and none of the mice died from injections with 10^7^ CFU, similar to Listeria alone (Supplementary Table 2). Finally, the same attenuated Listeria strain has been used for almost a decade in immune therapeutic approaches in cancer patients (delivering tumor-associated antigens into antigen-presenting cells), and it was demonstrated that Listeria is safe and non-toxic [20, 21]. Also ^32^P has been used (at much higher doses than in the current study) for more than 50 years in the clinic to treat polycythemia vera (a hematologic disorder) [22]. Although side effects are known, such as early leucopenia and thrombocytopenia, they resolve spontaneously.

Of note is that Listeria-based vaccines have been tested pre-clinically and clinically [15, 23, 24]. However, these studies are based on a complete different principle, i.e. tumor-associated antigens were delivered into antigen-presenting cells, while in the current study anticancer agent ^32^P was selectively delivered by Listeria to the TME and into tumor cells, underlining the novelty of our study.

Targeted radionuclide therapy has proven successful in the treatment of several types of cancer through radiolabeled small molecules, monoclonal antibodies (Abs), peptides, and other tumor-targeting vehicles [25], but for pancreatic cancer very modest results both preclinically and in cancer patients with unresectable liver metastases has been shown [26–29]. Therefore, our results are remarkable, and strongly suggest that Listeria-^32^P has great promise for the treatment of pancreatic cancer. Moreover, Listeria-^32^P was even more effective than Listeria-^188^Re, most likely because of the longer half-life of ^32^P (14 days) compared to ^188^Re (16.9 hrs), without having serious side effects analyzed 1 and 3 months after 12 treatments with Listeria-^32^P in Panc-02 mice (Supplementary Tables 1 and 2). Clinically, Listeria-^32^P is highly attractive because of its simple production (no antibodies are required like with ^188^Re), and its minimal side effects compared to chemotherapy or radiation, and its remarkable effect on pancreatic cancer.

Bacterial therapies have the potential to transform the field of cancer treatment, particularly against metastatic and incurable cancers. Pilot studies and clinical trials with modified bacteria are published or underway. Examples are *Salmonella typhimurium* against metastatic melanoma [30, 31], *Clostridium butyricum* against vascular gliomablastoma [32], and Clostridium novyiNT against solid tumors (NCT01924689). Here we demonstrate that Listeria-^32^P is highly promising against pancreatic cancer, and a pilot study with Listeria-^32^P in PDAC patients is under investigation.

In conclusion, the results from this study strongly suggest that Listeria-^32^P might be particularly useful for unresectable and metastatic cancers, like pancreatic cancer, and may be for other cancers as well.

## MATERIALS AND METHODS

### Mice

Normal female C57BL/6 mice (3 months) were obtained from Charles River and KPC mice (LSL-p53R172/+; LSLKrasG12D; Pdx1-Cre) [33] were generated in the laboratory of Dr. Steven K Libutti. All mice were maintained in the animal husbandry facility of Einstein according to the Association and Accreditation of Laboratory Animal Care (AACAC) guidelines. All mice were kept under BSL-2 and RSL-1B as required for Listeria and ^32^P treatments.

### Cells and cell culture

The highly metastatic Panc-02 cell line was derived in 1984 from a methylcholanthrene-induced ductal adenocarcinoma growing in a C57BL/6 female mouse [34] (kindly provided by Chandan Guha, Department of Radiation Oncology, Albert Einstein College of Medicine, Bronx, NY). The Panc-02 cells were cultured in McCoy's medium supplemented with 10% FBS, Glutamine (2 mM), non-essential amino acids, sodium pyruvate (1 mM), Hepes (10 mM), and Pen/Strep (100 U/ml) [8].

### Listeria monocytogenes (attenuated)

In this study, a highly attenuated *Listeria monocytogenes* (Listeria) was used as the vehicle for the delivery of ^32^Phosphorus (^32^P) to the tumor microenvironment (TME) and was generated in previous studies [15, 35] Briefly, the Listeria plasmid, pGG-34, expresses the positive regulatory factor (prfA) and Listeriolysin O (LLO) [36]. prfA regulates the expression of other virulence genes, and is required for survival *in vivo* and *in vitro*. The background strain XFL-7 lacks the prfA gene, and retains the plasmid *in vitro* and *in vivo* [36]. The coding region for the C-terminal part of the LLO (cytolytic domain that binds cholesterol in the membranes) protein in the plasmid has been deleted, but Listeria is still able to escape host vacuole [37]. Mutations have been introduced into the prfA gene and the remaining LLO (expressed by the pGG34 vector), which further reduced the pathogenicity of the Listeria [37].

### Incorporation of ^32^P into Listeria bacteria and preparation for injection

Listeria bacteria (0.5 × 10^9^ CFU) were first starved in 1 ml of saline for 30 min at 37°C at 200 rpm, and then cultured in 1 ml of Edinburgh Minimal Media Phosphate Free (EMMP) medium (US Biologicals Life Sciences, Pittsburgh, PA; Catalog: E2205-20), complemented with 50 μCi of ^32^P in deionized water (Phosphorus-32 Radionuclide Orthophosphoric acid in water; Perkin Elmer, New Rochelle, NY; cat # NEX053S005MC), for 60 min at 37°C and 200 rpm. Subsequently, the Listeria-^32^P culture was centrifuged and resuspended in 1 ml of saline and diluted with saline (final concentration: 5 × 10^7^ CFU/ml); 200 μl was injected (intraperitoneally) per dose, which equals 10^7^ CFU of Listeria and 1 μCi of ^32^P. The number of radioactive counts per min was measured in the pellet and supernatant by a gamma counter (1282 CompuGamma CS Gamma Counter, LKB Wallac, Long Island Scientific, East Setauket, NY). To determine the incorporation efficiency of ^32^P into Listeria, the total number of radioactive counts in the pellet was divided by the total number of radioactive counts in the pellet plus supernatant × 100%.

### Dissociation grade of Listeria-32P

Listeria was incorporated with ^32^P as described above. Subsequently, a stock solution was made of 10^9^ CFU of Listeria-^32^P in 500 ul mouse serum and incubated at 37°C. At 0, 1, 2, and 3 hrs, 100 ul of the stock solution was centrifuged and the radioactivity was determined in the pellet and supernatant as described previously. The dissociation grade was determined as described previously [8], by dividing the radioactive counts in the supernatant with the radioactive counts in the supernatant plus pellet × 100%.

### Tumor challenge, treatments and analyses

Effect of Listeria-^32^P on pancreatic cancer in Panc-02 or KPC mice. For the Panc-02 model, C57Bl/6 mice were injected with 2 × 10^6^ or 10^5^ Panc-02 tumor cells, as indicated in the text, in the mammary fat pad as described previously [8]. On day 3 (early stage pancreatic cancer; tumors 1–2 mm) or day 10 (late stage pancreatic cancer; tumors of 8–10 mm), mice were injected with 10^7^ CFU of Listeria-^32^P (delivers 1 μCi of ^32^P), every 3 days for 14 days (total 6 doses), intraperitoneal (ip), or with 3 cycles of 4 doses of 10^7^ CFU of Listeria-^32^P and three days rest after each cycle (total 12 doses), respectively. 10^7^ CFU of Listeria, or ^32^P alone (1 μCi), or saline were used as the control groups. One week after the last treatment, mice were euthanized and analyzed for the frequency of metastases and tumor weight (in grams) as described previously [8). Briefly, metastases were visible by eye in the pancreas, clustered in area of the portal veins in the liver, in the mesenteric lymph nodes along the gastrointestinus (GI), and less frequently in the diaphragm, spleen and kidneys, while a small tumor develops in the membrane of the peritoneal cavity extended from the mammary fat pad (See Supplementary Figure 3). Counting was based on number and size. KPC mice were treated with 12 doses of Listeria-^32^P (late stage pancreatic cancer; tumors with a SUVmax: range 2.0–7.0 corresponding with 5–25 mm diameter, at the age of 4.5–5 months), similar as the Panc-02 mice with late stage pancreatic cancer. Effect of Listeria-^32^P was monitored before and after treatment by PET/CT or PET.

### Ascites production in the peritoneal cavity

At the end of treatments with Listeria-^32^P all mice with early or advanced pancreatic cancer were graded for the production of ascites in the peritoneal cavity of the Panc-02 mice by visual inspection. (KPC mice do not produce ascites because development of metastases in the liver is less aggressive in KPC than Panc-02 mice). The gradation of ascites production was graded from A0 to A5. A0 = no ascites production (0), A1= some ascites production (+), A2 = some to moderate ascites production (++), A3 = moderate ascites production (+++), A4 = moderate-strong ascites production (++++), and A5 = strong ascites production (+++++).

### Survival study

C57Bl/6 mice were injected with 2 × 10^6^ Panc-02 tumor cells as described above, and then treated with 12 doses of 10^7^ CFU of Listeria-^32^P or control groups, with 3 days rest after each cycle, and subsequently monitored according IACUC guidelines.

### PET/CT

The KPC mice were monitored for the presence of tumors and metastases by positron emission tomography (PET) using a radioactive tracer [18F] fluoro-2-deoxyglucose (FDG), as described elsewhere [38]. To visualize the organs we used computed Tomography (CT). The effect of treatment on tumor and metastases was determined by the standardized-uptake value (SUV) as described elsewhere [39]. The PET Acquisition was performed by Siemens INVEON Trimodal PET/SPECT/CT scanner, and then processed by IAW (Inveon Acquisition Workplace), and OSEM2D (Ordered Subset Expected Maximization 2D).

### Determination of Listeria and radioactive counts in tumor and normal tissues

C57Bl/6 mice were injected with 2 × 10^6^ tumor cells as described above, and 14 days later injected once with a high dose 0.5 × 10^7^ CFU of Listeria-^32^P or Listeria as indicated in the text. Mice were euthanized at various time points after the injection of Listeria-^32^P or Listeria, and metastases, tumors and normal tissues were dissected, weighted, and analyzed for the number of CFU of Listeria and gamma radiation in a gamma counter (Wallac, Turku, Finland) as described previously [8].

### Confocal microscopy

Ten-micrometer sections were cut from snap-frozen sections of tumor and normal tissues, stained with anti-Listeria antibodies followed by secondary antibody goat anti-rabbit IgG-Cy3-labeled, mounted with DAPI containing mounting medium (Vectashield), and analyzed by and Image J software as described previously [8].

### Pathological examination

Tissues were fixed in 10% buffered formalin, processed routinely for paraffin embedding, sectioned at 5 μm, and stained with hematoxylin and eosin (H&E). Each sample was evaluated via light microscopy (using a Zeiss Axioskop2) and photomicropgraphs were taken using a Zeiss AxioCam HRc with Axiovision software.

### Statistical analysis

To statistically analyze the effects of Listeria-^32^P on the growth of metastases and tumors in the pancreatic mice, the Mann-Whitney test was used. Values *p* < 0.05 were considered statistically significant. For the survival data, the Log-rank Mantel Cox test was used. Values *p* < 0.05 were considered statistically significant.

## SUPPLEMENTARY MATERIALS FIGURES AND TABLES



## References

[1] Olive KP, Jacobetz MA, Davidson CJ, Gopinathan A, McIntyre D, Honess D, Madhu B, Goldgraben MA, Caldwell ME, Allard D, Frese KK, Denicola G, Feig C (2009). Inhibition of Hedgehog signaling enhances delivery of chemotherapy in a mouse model of pancreatic cancer. Science.

[2] Dimou A, Syrigos KN, Saif MW (2012). Overcoming the stromal barrier: technologies to optimize drug delivery in pancreatic cancer. Ther Adv Med Oncol.

[3] Wang Z, Li Y, Ahmad A, Banerjee S, Azmi AS, Kong D, Sarkar FH (2011). Pancreatic cancer: understanding and overcoming chemoresistance. Nat Rev Gastroenterol Hepatol.

[4] Siegel RL, Miller KD, Jemal A (2015). Cancer statistics, 2015. CA Cancer J Clin.

[5] Hidalgo M (2010). Pancreatic cancer. N Engl J Med.

[6] Kulke MH, Blaszkowsky LS, Ryan DP, Clark JW, Meyerhardt JA, Zhu AX, Enzinger PC, Kwak EL, Muzikansky A, Lawrence C, Fuchs CS (2007). Capecitabine plus erlotinib in gemcitabine-refractory advanced pancreatic cancer. J Clin Oncol.

[7] Chandra D, Jahangir A, Quispe-Tintaya W, Einstein MH, Gravekamp C (2013). Myeloid-derived suppressor cells have a central role in attenuated Listeria monocytogenes-based immunotherapy against metastatic breast cancer in young and old mice. British journal of cancer.

[8] Quispe-Tintaya W, Chandra D, Jahangir A, Harris M, Casadevall A, Dadachova E, Gravekamp C (2013). Nontoxic radioactive Listeria(at) is a highly effective therapy against metastatic pancreatic cancer. Proc Natl Acad Sci USA.

[9] Ostrand-Rosenberg S, Sinha P (2009). Myeloid-derived suppressor cells: linking inflammation and cancer. Journal of immunology.

[10] Bayne LJ, Beatty GL, Jhala N, Clark CE, Rhim AD, Stanger BZ, Vonderheide RH (2012). Tumor-derived granulocyte-macrophage colony-stimulating factor regulates myeloid inflammation and T cell immunity in pancreatic cancer. Cancer cell.

[11] Tilney LG, Portnoy DA (1989). Actin filaments and the growth, movement, and spread of the intracellular bacterial parasite, Listeria monocytogenes. J Cell Biol.

[12] Kim SH, Castro F, Paterson Y, Gravekamp C (2009). High efficacy of a Listeria-based vaccine against metastatic breast cancer reveals a dual mode of action. Cancer research.

[13] Chandra D, Gravekamp C (2013). Myeloid-derived suppressor cells: Cellular missiles to target tumors. Oncoimmunology.

[14] Singh M, Quispe-Tintaya W, Chandra D, Jahangir A, Venkataswamy MM, Ng TW, Sharma-Kharkwal S, Carreno LJ, Porcelli SA, Gravekamp C (2014). Direct incorporation of the NKT-cell activator alpha-galactosylceramide into a recombinant Listeria monocytogenes improves breast cancer vaccine efficacy. Br J Cancer.

[15] Kim SH, Castro F, Gonzalez D, Maciag PC, Paterson Y, Gravekamp C (2008). Mage-b vaccine delivered by recombinant Listeria monocytogenes is highly effective against breast cancer metastases. British journal of cancer.

[16] Yu M, Tannock IF (2012). Targeting tumor architecture to favor drug penetration: a new weapon to combat chemoresistance in pancreatic cancer?. Cancer Cell.

[17] Samba-Louaka A, Pereira JM, Nahori MA, Villiers V, Deriano L, Hamon MA, Cossart P (2014). Listeria monocytogenes dampens the DNA damage response. PLoS Pathog.

[18] Racz P, Tenner K, Mero E (1972). Experimental Listeria enteritis. I. An electron microscopic study of the epithelial phase in experimental listeria infection. Laboratory investigation; a journal of technical methods and pathology.

[19] Porembka MR, Mitchem JB, Belt BA, Hsieh CS, Lee HM, Herndon J, Gillanders WE, Linehan DC, Goedegebuure P (2012). Pancreatic adenocarcinoma induces bone marrow mobilization of myeloid-derived suppressor cells which promote primary tumor growth. Cancer Immunol Immunother.

[20] Gravekamp C, Paterson Y (2010). Harnessing Listeria monocytogenes to target tumors. Cancer biology & therapy.

[21] Wood LM, Paterson Y (2014). Attenuated Listeria monocytogenes: a powerful and versatile vector for the future of tumor immunotherapy. Front Cell Infect Microbiol.

[22] Tennvall J, Brans B (2007). EANM procedure guideline for 32P phosphate treatment of myeloproliferative diseases. European journal of nuclear medicine and molecular imaging.

[23] Maciag PC, Radulovic S, Rothman J (2009). The first clinical use of a live-attenuated Listeria monocytogenes vaccine: a Phase I safety study of Lm-LLO-E7 in patients with advanced carcinoma of the cervix. Vaccine.

[24] Le DT Wang-Gillam A, Picozzi V, Greten TF, Crocenzi T, Springett G, Morse M, Zeh H, Cohen D, Fine RL, Onners B, Uram JN, Laheru DA (2015). Safety and Survival With GVAX Pancreas Prime and Listeria Monocytogenes-Expressing Mesothelin (CRS-207) Boost Vaccines for Metastatic Pancreatic Cancer. J Clin Oncol.

[25] Shah M, Da Silva R, Gravekamp C, Libutti SK, Abraham T, Dadachova E (2015). Targeted radionuclide therapies for pancreatic cancer. Cancer Gene Ther.

[26] Qu CF, Songl YJ, Rizvi SM, Li Y, Smith R, Perkins AC, Morgenstern A, Brechbiel M, Allen BJ (2005). In vivo and in vitro inhibition of pancreatic cancer growth by targeted alpha therapy using 213Bi-CHX.A”-C595. Cancer Biol Ther.

[27] Milenic DE, Garmestani K, Brady ED, Albert PS, Ma D, Abdulla A, Brechbiel MW (2005). Alpha-particle radioimmunotherapy of disseminated peritoneal disease using a (212)Pb-labeled radioimmunoconjugate targeting HER2. Cancer Biother Radiopharm.

[28] Gold DV, Karanjawala Z, Modrak DE, Goldenberg DM, Hruban RH (2007). PAM4-reactive MUC1 is a biomarker for early pancreatic adenocarcinoma. Clinical cancer research.

[29] Sultana A, Shore S, Raraty MG, Vinjamuri S, Evans JE, Smith CT, Lane S, Chauhan S, Bosonnet L, Garvey C, Sutton R, Neoptolemos JP, Ghaneh P (2009). Randomised Phase I/II trial assessing the safety and efficacy of radiolabelled anti-carcinoembryonic antigen I(131) KAb201 antibodies given intra-arterially or intravenously in patients with unresectable pancreatic adenocarcinoma. BMC cancer.

[30] Heimann DM, Rosenberg SA (2003). Continuous intravenous administration of live genetically modified salmonella typhimurium in patients with metastatic melanoma. J Immunother.

[31] Toso JF, Gill VJ, Hwu P, Marincola FM, Restifo NP, Schwartzentruber DJ, Sherry RM, Topalian SL, Yang JC, Stock F, Freezer LJ, Morton KE, Seipp C (2002). Phase I study of the intravenous administration of attenuated Salmonella typhimurium to patients with metastatic melanoma. J Clin Oncol.

[32] Heppner F, Mose JR (1978). The liquefaction (oncolysis) of malignant gliomas by a non pathogenic Clostridium. Acta Neurochir (Wien).

[33] Hingorani SR, Wang L, Multani AS, Combs C, Deramaudt TB, Hruban RH, Rustgi AK, Chang S, Tuveson DA (2005). Trp53R172H and KrasG12D cooperate to promote chromosomal instability and widely metastatic pancreatic ductal adenocarcinoma in mice. Cancer cell.

[34] Corbett TH, Roberts BJ, Leopold WR, Peckham JC, Wilkoff LJ, Griswold DP, Schabel FM (1984). Induction and chemotherapeutic response of two transplantable ductal adenocarcinomas of the pancreas in C57BL/6 mice. Cancer Res.

[35] Pan ZK, Weiskirch LM, Paterson Y (1999). Regression of established B16F10 melanoma with a recombinant Listeria monocytogenes vaccine. Cancer research.

[36] Gunn GR, Zubair A, Peters C, Pan ZK, Wu TC, Paterson Y (2001). Two Listeria monocytogenes vaccine vectors that express different molecular forms of human papilloma virus-16 (HPV-16) E7 induce qualitatively different T cell immunity that correlates with their ability to induce regression of established tumors immortalized by HPV-16. J Immunol.

[37] Singh R, Dominiecki ME, Jaffee EM, Paterson Y (2005). Fusion to Listeriolysin O and delivery by Listeria monocytogenes enhances the immunogenicity of HER-2/neu and reveals subdominant epitopes in the FVB/N mouse. J Immunol.

[38] Yamamoto T, Seino Y, Fukumoto H, Koh G, Yano H, Inagaki N, Yamada Y, Inoue K, Manabe T, Imura H (1990). Over-expression of facilitative glucose transporter genes in human cancer. Biochem Biophys Res Commun.

[39] Nahmias C, Wahl LM (2008). Reproducibility of standardized uptake value measurements determined by 18F-FDG PET in malignant tumors. J Nucl Med.

