# Supplementary material for: 32-Phosphorus selectively delivered by listeria to pancreatic cancer demonstrates a strong therapeutic effect
Source: Oncotarget. 2017 Feb 6;8(13):20729–40. doi: 10.18632/oncotarget.15117 (PMC5400540; doi:10.18632/oncotarget.15117)
Supplement: Supplementary file 1 [file oncotarget-08-20729-s001.pdf]

32-Phosphorus selectively delivered by listeria to pancreatic cancer demonstrates a strong therapeutic effect

Supplementary Materials

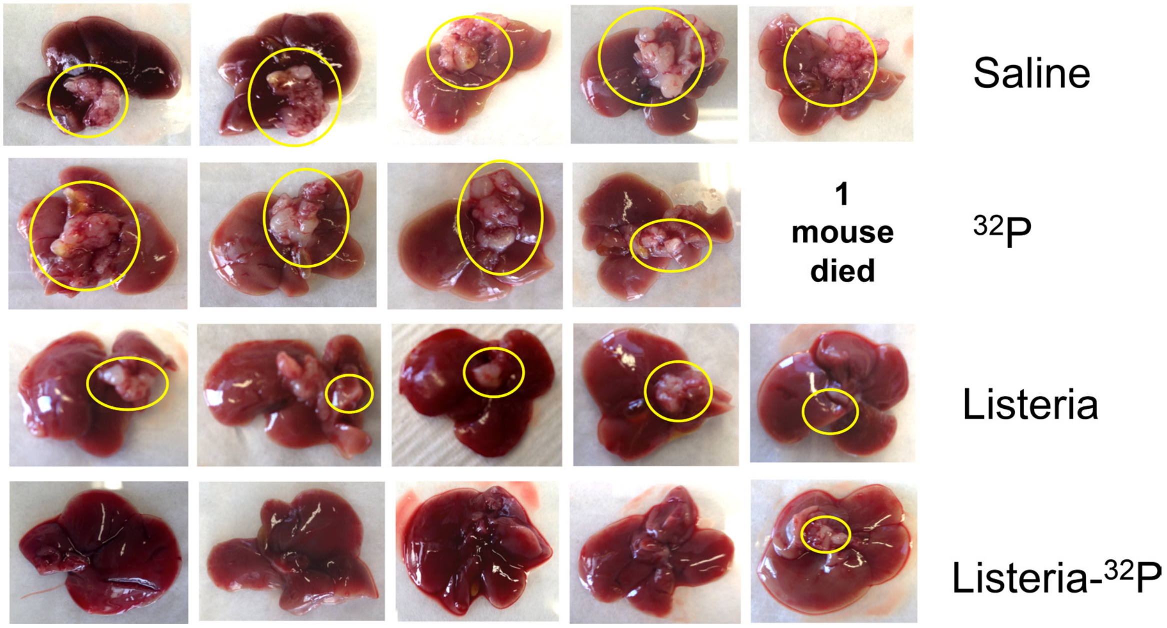

Supplementary Figure 1: Effect of Listeria-^{32}P on metastases in the portal liver of Panc-02 model. Here we show the effect of Listeria-^{32}P (LM-^{32}P), Listeria (LM), ^{32}P or saline on metastases in the portal liver of all five mice in one experiment. One mouse died in the ^{32}P alone group. Briefly, C57Bl/6 mice were injected with 2×10^6 Panc-0^2 tumor cells in the mammary fat pad. All untreated mice develop a primary tumor in the mammary fat pad that extends into the peritoneal membrane and is palpable 3–7 days after tumor cell injection (not shown), and metastases (visible by eye) develop predominantly in the pancreas, portal liver, and in the mesenteric lymph nodes (MLN). All untreated mice develop ascites and die around day 30 as described previously (1). On day 3, mice were injected with 10^7 CFU of LM-^{32}P (delivers 1 µCi of ^{32}P)(generated as indicated in the text and Figure 1A), or with 10^7 CFU of LM, or ^{32}P alone (1 µCi), or saline every 3 days for 14 days (6 doses in total), intraperitoneal (ip). One week after the last treatment (day 28), mice were euthanized and analyzed for metastases in the portal liver.

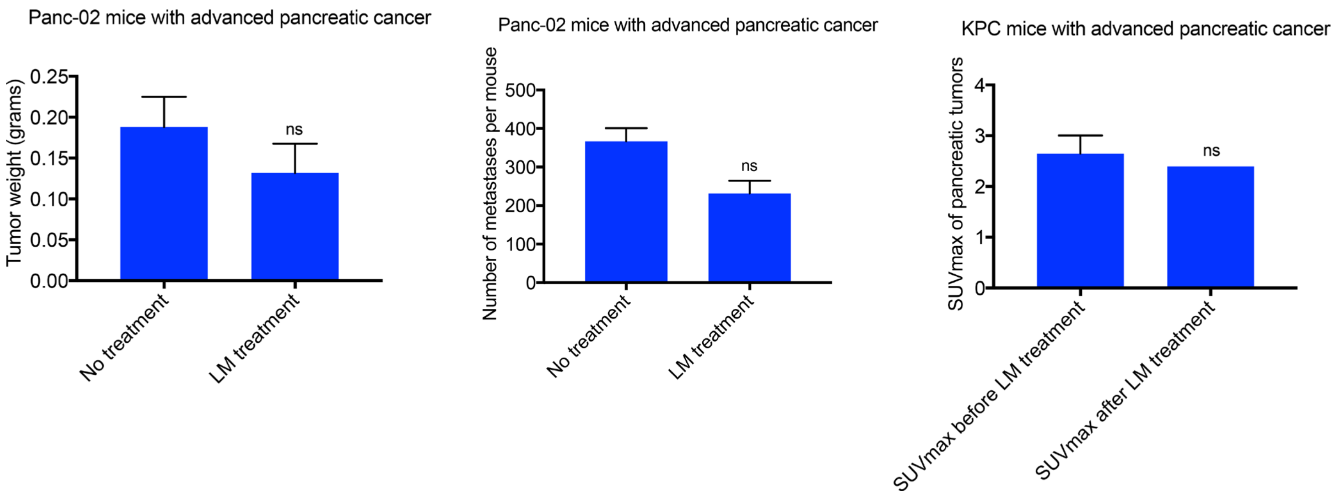

Supplementary Figure 2: Listeria alone has no significant effect on advanced pancreatic cancer in Panc-02 and KPC mice. (**A**) C57Bl/6 mice were injected with 10^5 Panc-02 tumor cells in the mammary fat pad. When tumors reached 8–10 mm (10 days after tumor cell injection), and metastases had spread to all organs, the Panc-02 mice received three cycles of 10^7 CFU of Listeria (LM) alone on four consecutive days, followed by a rest period of three days after each cycle (12 doses total). The mice were euthanized 6 weeks after tumor cell injection. $n = 5$ mice. The results were averaged. This experiment was performed once. Mann-Whitney $p < 0.05$ is significant. The error bars represent the SEM. (**B**) KPC mice with advanced pancreatic cancer (8 months old) received the same treatment with Listeria alone as the Panc-02 mice. The effect of Listeria treatment was analyzed by PET/CT before and at the end of the 12 treatments, expressed in SUVmax. $N = 3$ mice. The results were averaged. This experiment was performed once. Mann-Whitney $p < 0.05$ is significant.

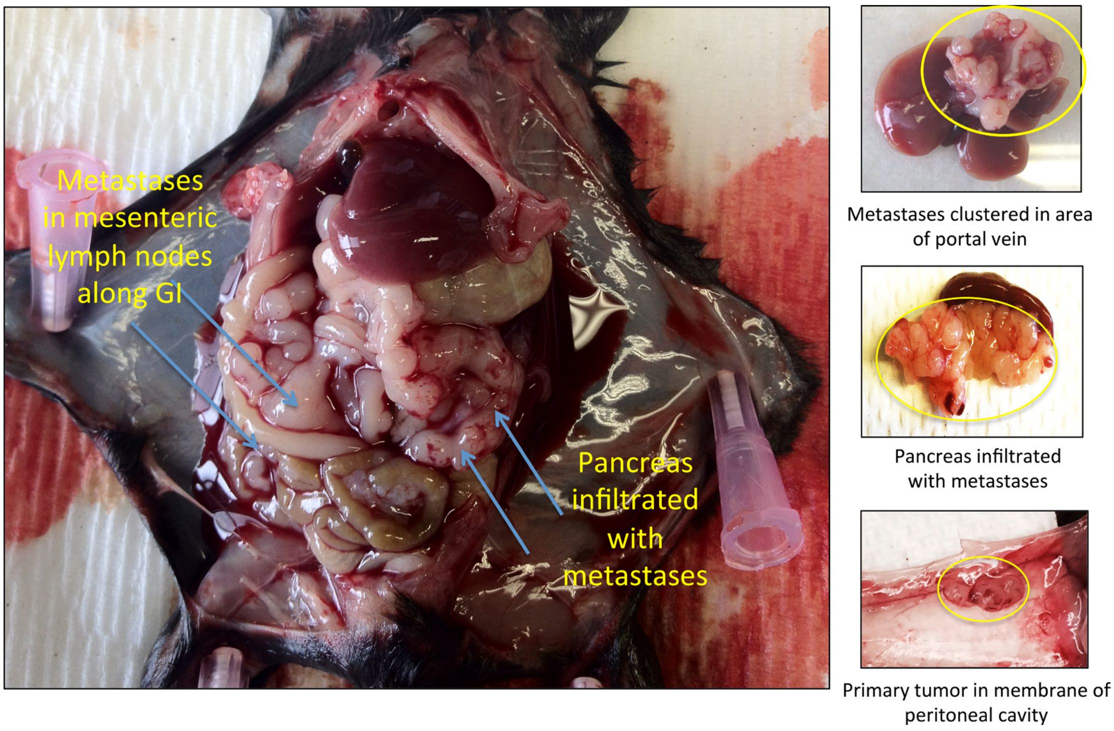

Supplementary Figure 3: Example of primary tumor and metastases in Panc-02 model. A primary tumor and metastases was generated as described previously (1). Briefly, C57Bl/6 mice were injected with 2×10^6 Panc-02 cells in the mammary fat pad. 3–7 days after injection a relatively small primary tumor is visible in the peritoneal membrane (extended from the mammary fat pad), and metastases are visible predominantly in the pancreas, liver, and mesenteric lymph nodes along the GI (and less frequently in diaphragm, spleen and kidneys; not shown). The picture of this highly aggressive model is shown 28 days after tumor cell injection.

REFERENCES

1. Quispe-Tintaya W, Chandra D, Jahangir A, Harris M, Casadevall A, Dadachova E, Gravekamp C. Nontoxic radioactive Listeria(at) is a highly effective therapy against metastatic pancreatic cancer. Proc Natl Acad Sci USA. 2013; 110:8668–73.

Supplementary Table 1: Pathology examination of tissues in mice without tumors (C57Bl/6) one month after last treatment with Listeria-^{32P} (LM-^{32P})

Treatment	LM-^{32P} (Group I)					Saline (Group II)				
**Mouse**	**m1**	**m2**	**m3**	**m4**	**m5**	**m1**	**m2**	**m3**	**m4**	**m5**
**Liver**										
Extramedullary hematopoiesis erythroid	1	1	1	0	0	0	0	0	0	1
Vacuolation cytoplasmic hepatocellular centrilobular pattern	0	0	0	0	0	0	0	0	0	0
Vacuolation cytoplasmic hepatocellular diffuse pattern	1	1	1	1	1	1	1	1	1	1
Individual hepatocyte cell death	0	0	0	0	0	0	0	0	0	0
Coagulation necrosis acute	0	0	0	0	0	0	0	0	0	0
Vascular thrombosis acute focal with perivascular inflammation	0	0	0	0	0	0	0	0	0	0
**Kidney**										
Tubule medullary dilated	0	1	0	0	0	0	0	0	0	0
**Spleen**	NSF	NSF	NSF	NSF	NSF	NSF	NSF	NSF	NSF	NSF
**Bone marrow**										
Bone marrow cellularity increased	NSF	NSF	NP	NSF	NSF	NSF	NSF	NSF	NSF	NSF
**Bone**										
No significant findings	NSF	NSF	NSF	NSF	NSF	NSF	NSF	NSF	NSF	NSF

Mice without tumors received 10^7 of CFU Listeria-^{32P} (LM-^{32P})(Group I) or saline (Group II) every 3 days for 2 weeks. $n = 5$ mice per group. Pathological examination was performed one month after the last treatment. The samples were graded on a scale of 0–5; 0 = no significant findings, 1 = minimal finding, 2 = mild finding, 3 = moderate finding, 4 = moderate-marked finding, 5 = marked finding, *P* = present, NP = not present, NSF = no significant finding. There are no notable findings in any of the samples evaluated. There were scattered multifocal minimal inflammation and extramedullary hematopoiesis in the liver, which is normally seen in mice and is not interpreted to be related to the treatment.

Supplementary Table 2: Pathology examination of tissues in Panc-02 mice treated with Listeria-^{32P} or Listeria and still alive and healthy at the end of the survival study

	LM-32P (Group I)			LM (Group II)
**Mouse**	**m1**	**m2**	**m3**	**m1**
**Kidney**	Ø	Ø	Ø	Ø
**Lymph node**	Ø	NP	NP	NP
**Liver**				
Inflammation, focal, mixed	1	2	1	0
Extramedullary hematopoiesis	0	1	0	0
Billary hyperplasia	0	1	0	0
**Small intestine**	Ø	Ø	Ø	Ø
Post mortem damage	3	3	5	4
**Spleen**				
Hyperplasia lymphodid germinal center	0	3	1	0
Plasmacytosis	0	2	0	0
**Heart**	Ø	Ø	Ø	Ø
**Lung**	Ø	Ø	Ø	Ø
**Bone**	NA	Ø	NA	Ø

At the end of the survival study (Figure 4c), 3 out of 5 mice that received LM-^{32P} were still alive and healthy 98 days later, and 1 out of 5 mice that received LM. These mice were examined for cancer and for treatment-induced pathological damage. 0 = no significant findings. 1 = minimal finding, 2 = mild finding, 3 = moderate finding, 4 = moderate to marked finding, 5 = marked finding, NP = tissues not present, NA = not applicable. Ø = no lesions in tissues.
